# Genetic Differentiation of Reintroduced Père David’s Deer (*Elaphurus davidianus*) Based on Population Genomics Analysis

**DOI:** 10.3389/fgene.2021.705337

**Published:** 2021-09-07

**Authors:** Shumiao Zhang, Chao Li, Yiping Li, Qi Chen, Defu Hu, Zhibin Cheng, Xiao Wang, Yunfang Shan, Jiade Bai, Gang Liu

**Affiliations:** ^1^Beijing Milu Ecological Research Center, Beijing, China; ^2^Beijing Key Laboratory of Wetland Services and Restoration, Institute of Wetland Research, Chinese Academy of Forestry, Beijing, China; ^3^College of Ecology and Nature Conservation, Beijing Forestry University, Beijing, China

**Keywords:** Père David’s deer, reintroduction, genetic differentiation, demographic history, population genomics

## Abstract

The reintroduction is an important conservation tool to restore a species in its historically distribution area, but the rate of reintroduction success varies across species or regions due to different reasons. Genetic evaluation is important to the conservation management of reintroduced species. Conservation concerns relate to genetic threats for species with a small population size or severely historically bottle-necked species, such as negative consequences associated with loss of genetic diversity and inbreeding. The last 40years have seen a rapid increasing of population size for Père David’s deer (*Elaphurus davidianus*), which originated from a limited founder population. However, the genetic structure of reintroduced Père David’s deer has not been investigated in terms of population genomics, and it is still not clear about the evolutionary history of Père David’s deer and to what extent the inbreeding level is. Conservation genomics methods were used to reconstruct the demographic history of Père David’s deer, evaluate genetic diversity, and characterize genetic structure among 18 individuals from the captive, free-ranging and wild populations. The results showed that 1,456,457 single nucleotide polymorphisms (SNPs) were obtained for Père David’s deer, and low levels of genome-wide genetic diversity were observed in Père David’s deer compared with Red deer (*Cervus elaphus*) and Sika deer (*Cervus nippon*). A moderate population genetic differentiation was detected among three populations of Père David’s deer, especially between the captive population in Beijing Père David’s deer park and the free-ranging population in Jiangsu Dafeng National Nature Reserve. The effective population size of Père David’s deer started to decline ~25.8ka, and the similar levels of three populations’ LD reflected the genetic impacts of long-term population bottlenecks in the Père David’s deer. The findings of this study could highlight the necessity of individual exchange between different facilities, and genetic management should generally be integrated into conservation planning with other management considerations.

## Introduction

The reintroduction has been increasingly used as an important and effective conservation tool to recover locally extirpated species, and a common tool for restoring lost biodiversity (Earnhardt, 1999; Armstrong and Seddon, 2008; Stewart et al., 2017). The rate of reintroduction success varies across species and differs from regions, and generally poor performance of reintroduction is reported due to different factors (Robert, 2009; Mowry et al., 2015; Robert et al., 2015; Thévenin, 2019). Genetic diversity is important to the viability and long term persistence of reintroduced species (Frankham, 2005; Steiner et al., 2012; Wirtz et al., 2018). In theory, the source of reintroduced populations is limited because of a small population size of founder individuals, which would result in a low level of genetic diversity and limited gene flow (Stewart et al., 2017; Ovenden et al., 2019). This means it is necessary to conduct population genetics after reintroduction, however, such post-reintroduction evaluation is unfortunately inadequate (Moseby et al., 2020), and genetic consequences following wild release still remain unknown for many cases (La Haye et al., 2017).

A key challenge of reintroduction efforts is to translocate individuals in a way that prevents loss of genetic variation, and avoids genetic differentiation relative to source populations (Barbanti et al., 2019). Such a challenge would have intensified, especially for a species that has recovered from a remnant population with historically low levels of genetic variation (Malone et al., 2018). Genetic diversity available for reintroduced populations is determined by the genetic background of the source population, which in turn is heavily impacted by the demographic history (Aspi et al., 2006). Conservation concerns relate to genetic threats for species with a small population size or severely historically bottle-necked species, such as negative consequences associated with loss of genetic diversity and inbreeding (Haig et al., 1990; Ewen et al., 2012). Genetic management plays an important role in improving the performance of reintroduction actions (Theodorou and Couvet, 2010). Several studies have evaluated genetic consequences induced by reintroduction events for some ungulates using traditional molecular markers, for example, Przewalskii’s wild horse (*Equus ferus*; Liu et al., 2014), European bison (*Bison bonasus*; Olech and Perzanowski, 2002), and Arabian oryx (*Oryx leucoryx*; El Alqamy et al., 2012), but only very few studies use genomics data to evaluate post-reintroduction genetic consequences (Flesch et al., 2020).

The unbiased genome-wide estimates generally reflect more accurately levels of genetic diversity and inbreeding (Kardos et al., 2016). The recent advances of next-generation sequencing (NGS) makes it feasible to detect genomic variations in many nonmodel species, and a new discipline, conservation genomics, is spawned in the new era, and is now expanding among conservation biologists (Angeloni et al., 2012; Xue et al., 2015; Wright et al., 2020; Hohenlohe et al., 2021). By taking a conservation genomics approach, genome-wide genetic diversity can be estimated in terms of neutral and non-neutral variation (Angeloni et al., 2012; Liu et al., 2020). In addition, genetic structure can be distinguished at a fine scale, even though the population differentiation is subtle (Lucek et al., 2019). Genomics data are useful for reintroduction projects by offering action guidance, including selecting source populations to maximize overall genomic variation, and valuable information for effective post-reintroduction monitoring (Latch, 2020). Meanwhile, the genomics data obtained also are important resources with the power to generate novel insights into the evolution of the focused species (Hohenlohe et al., 2021).

**Figure 1:**
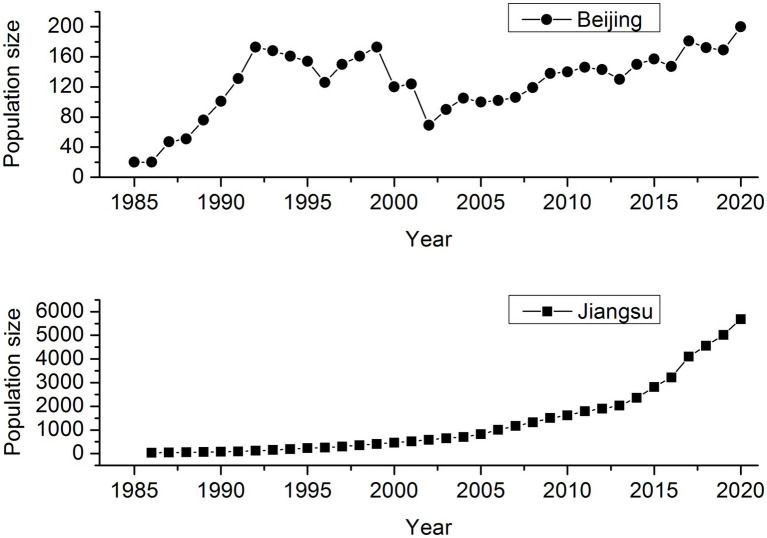
Population size trend of Père David’s deer in Beijing Père David’s deer Park and Jiangsu Dafeng National Nature Reserve (including both the free-ranging and wild populations).

Père David’s deer (*Elaphurus davidianus*), called “Milu” in China, went extinct in the wild as a result of over-hunting and habitat degradation in the late 19th century, but a few remnants were introduced into Europe and preserved safely in captivity in several European zoos. The remaining 18 individuals distributed in Berlin, Paris, and Antwerp were collected by the 11th Duke of Bedford, forming a breeding herd at the Woburn Abbey, England, where only 11 individuals were reported to have the ability to reproduce. By 1945, the global population size of Père David’s deer reached 250. Three reintroduction attempts were made in China. The first 20 deer were reintroduced to Beijing, China from Woburn Abbey in 1985, and then 18 in 1987. The second reintroduction population was established in Dafeng, Jiangsu Province, through trans-locating 39 deer from seven British zoos in August 1986. The third trans-located program included 94 deer from Beijing to Shishou, Hubei Province by three batches in 1993, 1994, and 2002. As of 2020, the population size of worldwide Père David’s deer is around 10,000, and exceeds 8,000 in China, suggesting a dramatic recovery over the last 40years although after a severe bottleneck in the history (Figure 1). Père David’s deer in the reintroduction sites serves as a multi-species conservation umbrella, and plays a key functional role in wetland ecosystems.

In recent years, wildlife protection laws and regulations have been gradually improved in China, and more suitable habitats are significantly provided. Efforts to rescue and protect endangered species have achieved remarkable results, with Père David’s deer as a successful icon in wildlife reintroduction. Père David’s deer provides an ideal system to evaluate the genetic consequences of reintroduction. Previous studies using mitochondrial DNA and microsatellites indicated that Père David’s deer had extremely low genetic diversity (Zeng et al., 2007, 2013). A comparative genomics study revealed that Père David’s deer was characterized with extensive genetic diversity (Zhu et al., 2018). However, genetic diversity and genetic structure have so far not been detected for the reintroduced populations of Père David’s deer at the genome-wide scale, and it is still not clear to what extent the inbreeding level is.

We used the genomics approach to reconstruct the demographic history of Père David’s deer, and to evaluate genetic diversity, and to characterize genetic structure among captive, free-ranging, and wild populations. We also tested the hypothesis that the captive population in Beijing Père David’s deer park is differentiated from the populations in Jiangsu Dafeng National Nature Reserve.

## Materials and Methods

### Sample Collection

Blood samples from 10 Père David’s deer individuals (four males and six females), classified as the captive population (MC), were collected from Beijing Père David’s deer Park, and four samples (two males and two females) as the free-ranging population (MF) in Dafeng National Nature Reserve, Jiangsu Province, and another four samples (two males and two females) as the wild population (MW) in Dafeng National Nature Reserve, Jiangsu Province. Here, the free-ranging population indicates the animals live in a quite large fence, but can feed and breed naturally there. In contrast, the wild population survives and reproduces in the wild absolutely. The free-ranging and wild population was separated physically despite being in Dafeng National Nature Reserve. Blood samples were collected from two individuals of Red deer (*Cervus elaphus*) and two individuals of Sika deer (*Cervus nippon*) in Beijing Père David’s deer Park. Blood samples were stored in vacuum blood collection tube (Heparin anticoagulation). All samples were collected in 2018 and 2019 (Supplementary Table 1).

### Sequencing and Single Nucleotide Polymorphism Calling

Genomic DNA was extracted using QIAamp DNA Blood Mini Kit (Qiagen, Germany). The libraries were prepared as described in stLRF kit, and whole-genome resequencing was performed based on the BGISEQ-500 platform (BGI). The adapter and low quality reads were removed by SOAPnuke (Chen et al., 2017). Clean reads were aligned against the reference genome of Père David’s deer (Project ID: PRJNA391565, downloaded from NCBI; Zhang et al., 2017) using Burrows-Wheeler Aligner (BWA; Li and Durbin, 2010), and then read alignments were sorted using SAMTools with the following parameters: “-q 1 -C 50 -g -t DP, SP, DP4 -I -d 250 -L 250 -m 2 -p,” and duplicate reads were marked by Picard.

Multisample single nucleotide polymorphism (SNP) genotyping were carried out as follows. Variants calling was conducted using HaplotypeCaller and GenotypeGVCFs in GATK4 (Van der Auwera et al., 2013), and in order to ensure the identification accuracy, SNPs were removed if any of the following parameter was met: QD<2, MQ<30, MQRankSum<−12.5, FS>200, ReadPosRankSum<20, QUAL<30.0, and AN<40. The GVCF file from each sample was generated using HaplotypeCaller, and then GenotypeGVCFs were used to merge separate GVCF files with the aim to improve the sensitivity of mutation detection.

### Genetic Diversity Estimation

In order to estimate the genetic diversity of reintroduced populations of Père David’s deer, VCFtools (Danecek et al., 2011) was used to calculate the nucleotide diversity *π* at the whole-genome scale for each population. The Watterson’s estimator (w) of each population was calculated using VariScan v2.0.3 (Hutter et al., 2006). Genome-wide Tajima’s *D* values for each population were calculated using VCFtools. A widely used approach for detecting selection is to apply a neutrality test statistic based on allele frequencies, with Tajima’s D being the most frequently one (Korneliussen et al., 2013).

### Population Structure Analysis

The population structure was mainly revealed through three different methods, including principal component analysis (PCA), phylogenetic tree (NJ tree), and ancestral component analysis (fastSTRUCTURE). Based on the filtered set of 1,456,457 SNPs, we used Genome-wide Complex Trait Analysis (GCTA; Yang et al., 2011) to perform principal component analysis (PCA), filter a few principal components through a dimensionality reduction algorithm, and use these principal components. Components can be used to explain the differences between individuals to the greatest extent, and at the same time, the clustering relationship of each sample based on the distribution of PCs can be estimated. A phylogenetic tree was constructed using Neighbor-joining (NJ) in Tree Building guided by Species Tree (TreeBeST; Price et al., 2010) in order to study the genetic distance relationship between individuals, with a bootstrap value of 1,000. The population genetic structure was inferred from genomics data using STRUCTURE with default settings (Raj et al., 2014). The most likely value of *K* was identified from 20 independent runs for each value of *K* ranging from 2 to 8, with 100 bootstraps. The posterior probability K was estimated, and the log likelihood was used to choose the optimal *K* (Evanno et al., 2005). The web-based Structure Harvester (Earl and vonHoldt, 2012) was used to assess and visualize likelihood values across multiple values of *K*. The genetic differentiation index, F_ST_ was usually used to detect the genetic composition variance between all populations, and we used VCFtools to calculate these values. The level of genetic differentiation can be defined as low (F_ST_<0.05), moderate (0.05<F_ST_<0.15), high (0.15<F_ST_<0.25), and extremely high (F_ST_>0.25; Wright, 1978).

### Demographic History and Inbreeding Pattern

Multiple sequential Markovian coalescence (MSMC) model (Schiffels and Durbin, 2014) can be used to reconstruct each population’s demographic history based on genomic information. MSMC breaks through the limitation that PSMC can only analyze one sample at a time. In addition, MSMC can integrate and analyze the nearest common ancestor time between multiple allele sequences at the same time, thereby improving the accuracy and efficiency of effective population size (Ne) prediction (Schiffels and Durbin, 2014). The parameters used were as follows: “-t 6 -p 1*2+15*1+1*2.” A mutation rate (*μ*) of 1.5×10^−8^ mutations per site per generation for the three Cervidae species in this study was according to the reference (Chen et al., 2019). The generation time was estimated to be 6years based on the report of mammal generation length (Pacifici et al., 2013). Detection of linkage disequilibrium (LD) patterns is necessary to infer population historical changes as well as inbreeding events in the species evolutionary history (Karimi et al., 2020). To assess the genomic extent of inbreeding inPère David’s deer, genome-wide LD was estimated for the three populations using PopLDdecay (Zhang et al., 2019). To assess the LD of Père David’s deer, the correlation coefficient (*R*^2^) between any two loci from each population was calculated using vcftools v0.1.14, with following parameters: “--ld –window -bp 500,000 –geno -r2,” and average *R*^2^ values were calculated for pairwise SNPs by keeping the same distance.

## Results

### Genetic Diversity and Genetic Drift

A total of 12,744G clean reads were obtained among 22 samples from Père David’s deer, Red deer, and Sika deer (Supplementary Table 1). The results showed that the average genome coverage rate was 98.77%, and the average sequencing depth was 19.93X, with an average mismatch rate of 0.90% (Supplementary Table 2). After filtering, an average of 485.76 million clean reads remained for each Père David’s deer sample, with a clean data ratio of 99.31%, of which 99.12% reads (98.18–99.67%) were mapped to the Père David’s deer reference genome, and the mean GC content was 43.33%. Totally, 1,456,457 SNPs were obtained for all the datasets for further analysis, which had 33.92% homozygotes and 66.08% heterozygotes, meaning a heterozygosity rate of 0.38 per kilobase pair in the Père David’s deer (Supplementary Figure 1). The heterozygote percentage of the free-ranging and wild populations of Père David’s deer (70.47±0.81%) was significantly higher than the captive population (64.21±0.76%; *p*<0.01). Across all 18 samples from Père David’s deer, 367.266 indels were identified, with 54.61% insertions and 45.39% deletions, but neither the percentage of insertion nor deletion showed significant difference across Père David’s deer populations (*p*>0.05).

Whole genome variation based on re-sequencing revealed that free-ranging Père David’s deer (MF) had the highest genomic diversity (*π*=4.03×10^−5^, *w*=3.58×10^−5^), whereas captive individuals (MC) had the lowest genetic diversity (*π*=4.03×10^−5^, *w*=2.99×10^−5^). Père David’s deer had lower genetic diversity than Red deer (*π*=1.04×10^−4^, *w*=1.04×10^−4^) and Sika deer (*π*=1.47×10^−4^, *w*=1.45×10^−4^; Table 1). The genome-wide Tajima’s *D* values were positive in three populations of Père David’s deerr (Table 1), indicating that Père David’s deer experienced balancing selection and population contraction in the history.

**Table 1:** The genetic diversity index for three populations of Père David’s deer and its related species.

	π	w	Tajima’s D
MC	0.0000403	0.0000299	0.4908771
MF	0.0000409	0.0000358	0.2367667
MW	0.00004	0.0000351	0.2300057
RD	0.0001041	0.0001037	0.0074172
SK	0.0001466	0.0001452	0.0213304

MC, captive Père David’s deer; MF, free-ranging Père David’s deer; MW, wild Père David’s deer; RD, Red deer; and SK, Sika deer.

### Population Genetic Structure

**Figure 2:**
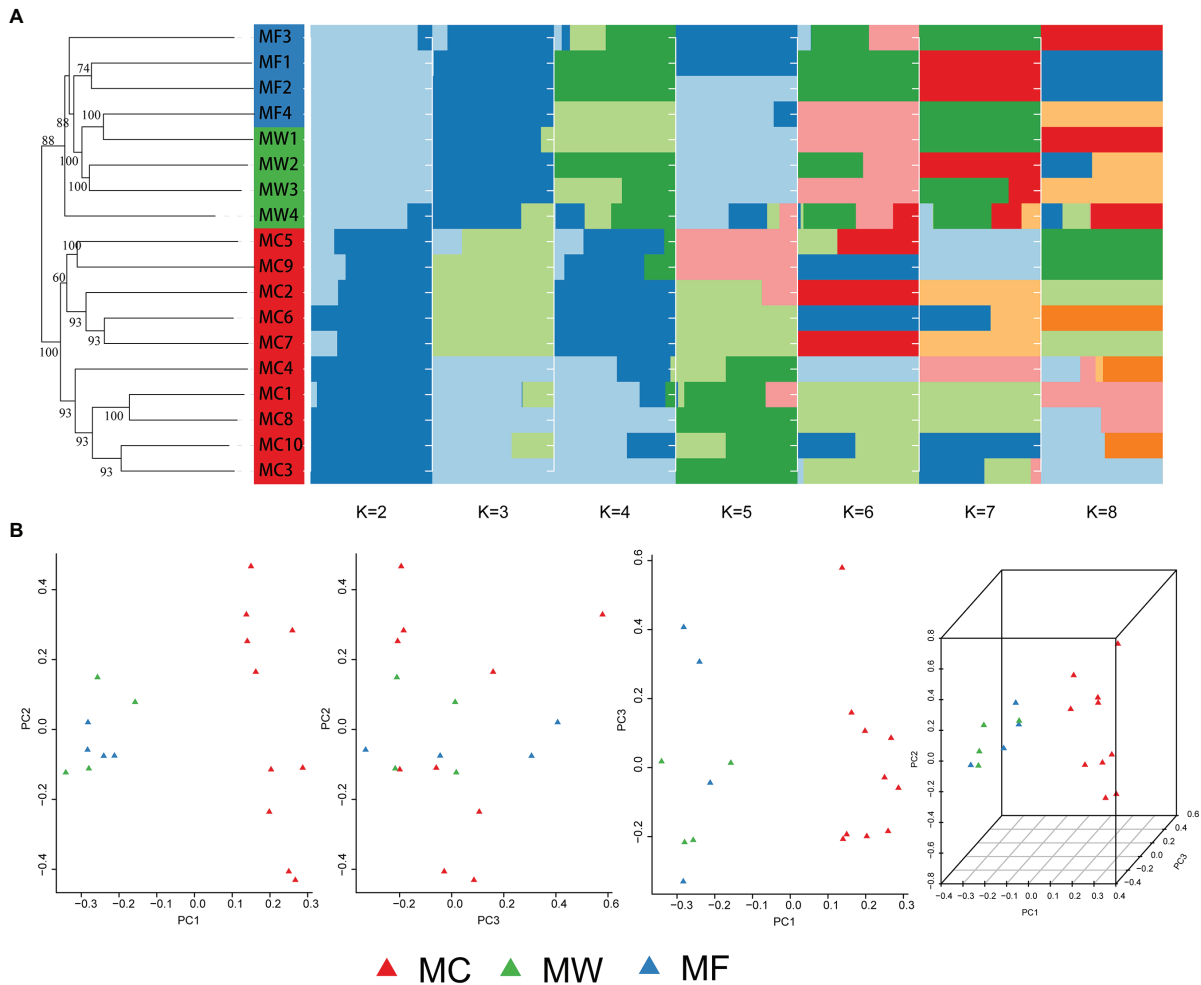
Population genetic structure among the captive population (MC), the free-ranging population (MF), and the wild population of reintroduced Père David’s deer. **(A)** Neighbor-joining (NJ) phylogenetic tree (left) and subgroups represented by the STRUCTURE analysis (*K*=2, 3, 4, 5, 6, 7, and 8 shown; right). **(B)** Principle Component Analysis (PCA) plot.

The captive population of Père David’s deer from Beijing Milu Park showed moderate population genomic differentiation from free-ranging and wild populations from Jiangsu based on both STRUCTURE and PCA analysis. Within the populations from Jiangsu, free-ranging and wild populations were further divided into two genetic subgroups, but population admixture also existed (Figure 2). The individuals from MF population admixed with MW population, and the individuals from MW population also clustered into MF population. This genetic assignment implied relatively frequent gene flow between individuals within the reintroduced population in Jiangsu. At a cluster value of two (*K*=2; Supplementary Figure 2), STRUCTURE performed well to distinguish among three populations of Père David’s deer, suggesting the reintroduced populations had separated into two genetic structures (Figure 2A). The NJ phylogenetic tree based on pairwise SNP differences also revealed separate genetic clusters among captive, free-ranging, and wild populations of the reintroduced populations (Figure 2A). The average *F*_ST_ value between the captive and free-ranging was 0.073906, and 0.07302 between the captive and wild populations, suggesting a moderate genetic differentiation (Table 2). The average *F*_ST_ value was negative between the free-ranging and wild population, indicating no genetic differentiation, which was consistent with the results generated by PCA (Figure 2B) and phylogenetic tree.

**Table 2:** The genetic differentiation index between populations base on F_ST_.

	SK	RD	MW	MF	MC
MC	0.910565	0.91975	0.07302	0.073906	0
MF	0.888112	0.904536	−0.00976	0	
MW	0.889444	0.905702	0		
RD	0.586584	0			
SK	0				

MC, the captive population; MF, the free-ranging population; MW, the wild population; RD, Red deer; and SK, Sika deer.

### Demographic History

**Figure 3:**
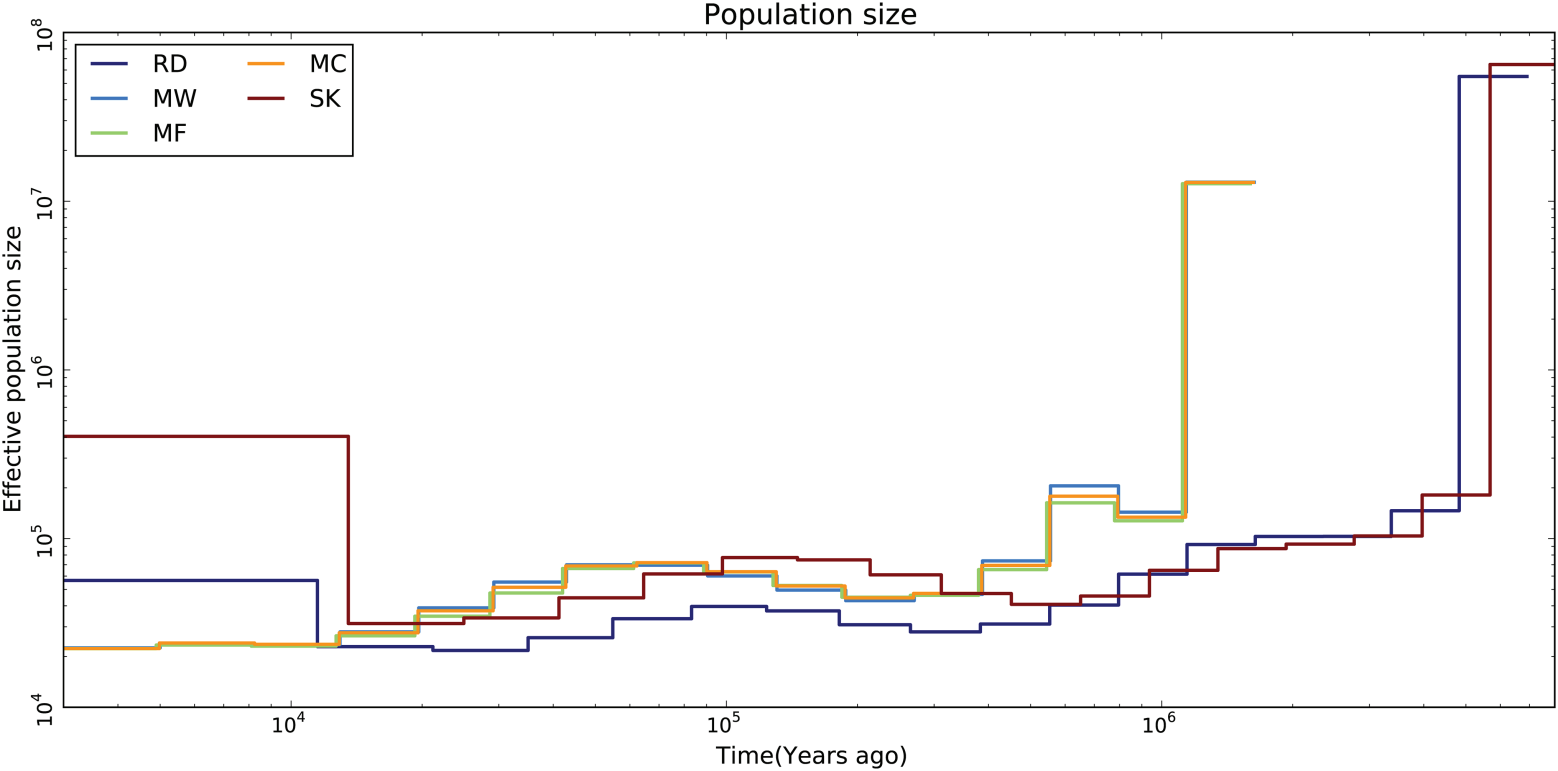
Demographic history of Père David’s deer populations (MC, MF, and MW), Red deer (RD) and Sika deer (SK). Effective population size by time was estimated using MSMC2.

The results from the MSMC analysis showed that the effective population size of Père David’s deer started to decline ~25.8ka. The three populations of Père David’s deer had a similar long-term demographic history. About 2.58×10^4^years ago, the effective population sizes for Père David’s deer were always larger than those for Red deer and Sika deer, but over the past 1.25×10^4^years, it showed an opposite trend (Figure 3).

### Inbreeding Pattern

**Figure 4:**
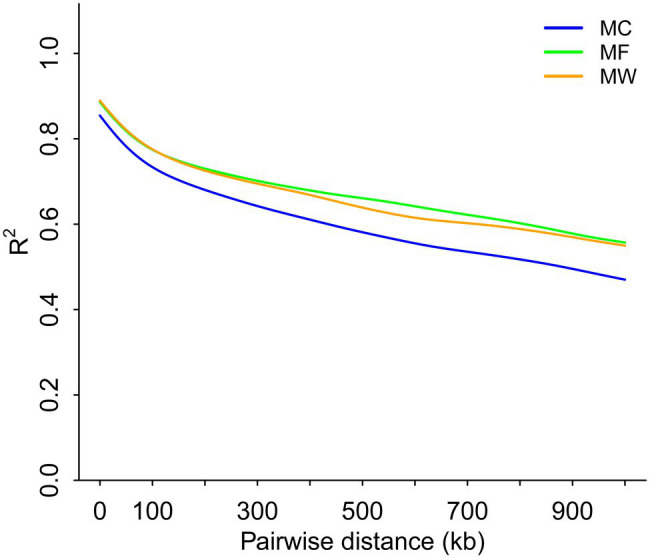
Whole-genome scale patterns of linkage disequilibrium across reintroduced Père David’s deer populations.

Genome-wide LD analysis demonstrated that the average pairwise distance for the LD to decay until *R*^2^=0.4 was >900kb for Père David’s deer (Figure 4). The three populations of Père David’s deer had similar levels of LD, to some extent reflecting the genetic impacts of long-term population bottlenecks in the Père David’s deer.

## Discussion

This is, to our knowledge, the first study to evaluate the genomic consequences of reintroduced Père David’s deer using the approach of population genomics, covering captive, free-ranging, and wild populations. The genome-wide genetic diversity of Père David’s deer was lower than that of Red deer and Sika deer. This result was consistent with other earlier studies using mitochondrial DNA and nuclear DNA (Zeng et al., 2007), and microsatellite (Wu et al., 2008; Zhang et al., 2010). In addition, the captive population had the lowest genomic diversity, while the highest was revealed in the free-ranging population. Although 18 individuals were considered as the founder source of all current populations of Père David’s deer around the world, 13 of them contributed to the breeding from the start at the Woburn Abbey, England. Père David’s deer underwent drastic declines in population size, during which population decline is always accompanied by loss of genomic diversity due to genetic drift and inbreeding. Furthermore, the 12th Duke of Bedford even suspected that all current populations of Père David’s deer were descended from a single male (Sowerby, 1949). Up to now, unfortunately almost nothing is known about the composition and structure of the Père David’s deer Y chromosome, and further knowledge on the Y chromosome in future studies may be crucial for adding a new dimension to our understanding of the low level of genomic diversity. Taking the Przewalski’s horse as an example, only two haplotypes of Y chromosome were kept after experiencing a severe historically bottleneck (Kavar and Dovc, 2008), and the limited Y chromosome lineages partially contribute to homozygous variations of Przewalski’s horse (Do et al., 2014).

To be noticed, the genomic diversity in the captive population was lower than that in the free-ranging population of Père David’s deer (Table 1), which was contrary to the results based on microsatellite (Zeng et al., 2007). Such inconsistent results may be attributed to different methods applied in different studies. Compared with neutral loci (e.g., microsatellite), the genomic approach can increase the power and accuracy of estimating a set of key parameters in conservation biology (Allendorf et al., 2010; Fan et al., 2018). Conservation breeding actions play an important role to protect species by controlling threatening factors and providing reintroduction source (Ebenhard, 1995). However, a key issue associated with captive breeding is the limited gene pool, leading to the accumulation of inbreeding from the founder generation, due to small population sizes and constrained migration (Alroy, 2015; Mulvena et al., 2020). The origin of Père David’s deer differs between Beijing and Jiangsu (Bai et al., 2012; Cao, 2021), which means that genetic composition varies between regions. In addition, small populations are expected to suffer from loss of genetic diversity through genetic drift and reduced fitness because of inbreeding (Kvie et al., 2019). As of 2000, the captive population size in Beijing has not exceeded 200 individuals, and in contrast, the population size in Jiangsu has nearly reached 6,000 (Figure 1).

A moderate genetic differentiation was represented among captive, free-ranging, and wild populations by both STRUCTURE and PCA analysis (Figure 2). The captive and free-ranging populations showed the highest level of genetic differentiation (Table 2), which was supported by the microsatellite based study (Zeng et al., 2007). The population in Beijing was reintroduced from the Woburn Abbey, but the population in Jiangsu was established with founders from five zoo populations (Jiang and Ding, 2011). The different lineage would be the main reason resulting in different genetic structure. In the past 40years, 520 Père David’s deer in Beijing Milu Park have been released into 40 sites through 53 translocation events covering 20 provinces around China, but unfortunately, few individuals were translocated to Dafeng, Jiangsu. The lack of translocation between Beijing and Jiangsu may hinder the gene flow, and further drive genetic differentiation.

The demographic history analysis based on MSMC2 showed that the effective population size of Père David’s deer started to decline ~25.8ka (Figure 3), which coincides with the inference according to PSMC (Zhu et al., 2018). During late Pleistocene, large mammal declines became much more severe than previously expected due to the interaction effects of climate change and human activities, although the impact varies across continents or species (Barnosky et al., 2004; Chen et al., 2019). With the human population expanding dramatically (Li and Durbin, 2011), the population decline of Père David’s deer might be at least partially attributable to human activities (e.g., human hunting), which was further supported by the fossil evidence (Dong et al., 2019). This is further supported by new evidence from large-scale ruminant genome sequencing, given that the demographic pattern was species-specific because of variable habitat types or feeding types (Chen et al., 2019). With the population size decreasing, inbreeding accumulated during the evolutionary history of Père David’s deer (Zhu et al., 2018). The LD pattern is a useful indicator for estimating inbreeding at the individual level, and generally LD rapidly decays to very low levels in populations with low inbreeding level (Wang et al., 2021). Père David’s deer exhibited extremely high LD (Figure 4), which implied that a prolonged population decline might have caused an increased overall burden of inbreeding.

## Conservation Implications

This study evaluates genomic consequences of post-reintroduction of Père David’s deer using the conservation genomics approach, and the results would contribute to inform future conservation management. There are many factors influencing species reintroduction success, among which genetic is the most important (Flesch et al., 2020). The genetic diversity of an endangered species is one of the main parameters that can directly reflect the evolutionary potential. Genetic evaluation can provide decision-making reference for the relevant government departments to formulate species protection strategies and plans. Genomic evaluation of reintroduced populations can help address the uncertainties in reintroduction projects. According to IUCN guidelines, genetic management should generally be integrated into conservation planning with other management considerations (Ralls et al., 2018).

In the future, the genetic evaluation should cover more populations and sample more individuals, for example, the population size of Père David’s deer has reached 1,600 in Shishou, Hubei province. The extremely low genetic diversity has always been a warning to humans that population genetic health of Père David’s deer is still relatively fragile, and how to maintain the current level and prevent further loss still remains a concern in conservation management. In the specific implementation of the reintroduction, there are still some geopolitical factors that may affect the source selection and the selection of reintroduction sites. It is recommended to conduct individual exchanges among different facilities, so as to enhance gene flow and reduce inbreeding. For example, the captive individuals in Beijing should be selected to be released to the wild in Jiangsu Dafeng National Nature Reserve. Before releasing, the genetic background of the source individuals should be monitored to ensure sufficient gene flow. In addition, the samples of Père David’s deer are important resources for scientific research, especially the ones who exhibited abnormal phenotypes in the population, because they can be used to study the inbreeding depression and genetic changes over time. So, we suggest building a sample library for reintroduced species.

## Data Availability Statement

The datasets presented in this study can be found in online repositories. The names of the repository/repositories and accession number(s) can be found at: https://www.ncbi.nlm.nih.gov/, Project ID: PRJNA756107.

## Ethics Statement

The animal study was reviewed and approved by China Wildlife Conservation Association.

## Author Contributions

SZ and GL conceived the study. YL, QC, ZC, XW, and YS performed the experiments. GL and CL performed the bioinformatics analyses and data analysis. DH and JB revised the manuscript. All authors contributed to the article and approved the submitted version.

## Funding

This study was funded by Innovation Team Program of Beijing Academy of Science and Technology “Research on Genetic Resources Conservation and Fine Management of Milu” [IG201805N/C1 (newly added)].

## Conflict of Interest

The authors declare that the research was conducted in the absence of any commercial or financial relationships that could be construed as a potential conflict of interest.

## Publisher’s Note

All claims expressed in this article are solely those of the authors and do not necessarily represent those of their affiliated organizations, or those of the publisher, the editors and the reviewers. Any product that may be evaluated in this article, or claim that may be made by its manufacturer, is not guaranteed or endorsed by the publisher.
