# Supplementary material for: Genetic Differentiation of Reintroduced Père David’s Deer (Elaphurus davidianus) Based on Population Genomics Analysis
Source: Front Genet. 2021 Sep 7;12:705337. doi: 10.3389/fgene.2021.705337 (PMC8452920; doi:10.3389/fgene.2021.705337)

Supplementary Material



**Supplementary Figure 1.** The homozygote and heterozygote percentage of each Père David's deer sample.

**Supplementary Table 1.** Sequencing data quality of Père David's deer populations (MC, MF and MW), Red deer (RD) and Sika deer (SK).

| **Sample** | **Raw_reads**  **(M)** | **Raw_bases**  **(Gb)** | **Clean_reads**  **(M)** | **Clean_bases**  **(Gb)** | **Clean_ratio**  **(%)** | **Q20**  **(%)** | **Q30**  **(%)** | **GC**  **(%)** |
| --- | --- | --- | --- | --- | --- | --- | --- | --- |
| MC1 | 632.57 | 63.26 | 630.66 | 63.07 | 99.70 | 98.42 | 94.97 | 43.46 |
| MC2 | 627.49 | 62.75 | 622.29 | 62.23 | 99.17 | 97.85 | 93.65 | 43.40 |
| MC3 | 610.13 | 61.01 | 605.01 | 60.50 | 99.16 | 97.88 | 93.70 | 43.46 |
| MC4 | 650.95 | 65.10 | 649.20 | 64.92 | 99.73 | 98.40 | 94.74 | 43.53 |
| MC5 | 597.46 | 59.75 | 595.86 | 59.59 | 99.73 | 98.46 | 95.09 | 43.57 |
| MC6 | 620.88 | 62.09 | 619.26 | 61.93 | 99.74 | 98.41 | 94.80 | 43.43 |
| MC7 | 640.53 | 64.05 | 639.12 | 63.91 | 99.78 | 98.50 | 94.91 | 43.49 |
| MC8 | 515.71 | 51.57 | 514.42 | 51.44 | 99.75 | 98.47 | 95.14 | 43.52 |
| MC9 | 711.08 | 71.11 | 709.45 | 70.94 | 99.77 | 98.48 | 95.00 | 43.61 |
| MC10 | 596.67 | 59.67 | 590.36 | 59.04 | 98.94 | 97.87 | 93.50 | 43.07 |
| MF1 | 565.43 | 56.54 | 559.68 | 55.97 | 98.98 | 97.59 | 92.90 | 42.67 |
| MF2 | 579.49 | 57.95 | 574.69 | 57.47 | 99.17 | 97.89 | 93.70 | 42.59 |
| MF3 | 540.18 | 54.02 | 534.34 | 53.43 | 98.92 | 97.61 | 93.00 | 42.82 |
| MF4 | 553.76 | 55.38 | 548.34 | 54.83 | 99.02 | 97.60 | 92.86 | 42.73 |
| MW1 | 622.02 | 62.20 | 615.21 | 61.52 | 98.91 | 97.78 | 93.49 | 42.63 |
| MW2 | 448.98 | 44.90 | 445.03 | 44.50 | 99.12 | 97.73 | 93.30 | 43.18 |
| MW3 | 478.13 | 47.81 | 473.53 | 47.35 | 99.04 | 97.78 | 93.36 | 43.24 |
| MW4 | 587.77 | 58.78 | 582.28 | 58.23 | 99.07 | 97.87 | 93.78 | 42.75 |
| SK1 | 541.54 | 54.15 | 540.33 | 54.03 | 99.78 | 98.51 | 95.07 | 44.97 |
| SK2 | 539.97 | 54.00 | 538.41 | 53.84 | 99.71 | 98.27 | 94.40 | 44.68 |
| RD1 | 623.32 | 62.33 | 616.65 | 61.67 | 98.93 | 97.97 | 93.90 | 44.14 |
| RD2 | 545.21 | 54.52 | 539.92 | 53.99 | 99.03 | 98.00 | 94.08 | 44.20 |

**Supplementary Table 2.** Alignment metrics of each sample from Père David's deer populations (MC, MF and MW), Red deer (RD) and Sika deer (SK).

| **Sample** | **Mapping**  **(%)** | **Unique Mapping (%)** | **Average depth**  **(X)** | **Mismatch**  **Rate (%)** | **Coverage**  **(%)** | **Coverage**  **(≥10X)**  **(%)** |
| --- | --- | --- | --- | --- | --- | --- |
| MC1 | 98.70 | 94.21 | 21.89 | 0.63 | 99.35 | 96.02 |
| MC2 | 99.39 | 94.27 | 21.56 | 0.76 | 99.54 | 93.87 |
| MC3 | 99.42 | 94.65 | 21.36 | 0.72 | 99.54 | 93.68 |
| MC4 | 98.39 | 94.04 | 22.39 | 0.66 | 99.37 | 94.69 |
| MC5 | 98.18 | 94.25 | 20.60 | 0.63 | 99.31 | 95.23 |
| MC6 | 98.60 | 94.18 | 21.50 | 0.64 | 99.28 | 95.74 |
| MC7 | 98.43 | 94.07 | 22.12 | 0.63 | 99.29 | 96.05 |
| MC8 | 98.60 | 94.03 | 17.93 | 0.66 | 99.29 | 90.27 |
| MC9 | 98.31 | 94.09 | 24.37 | 0.63 | 99.43 | 96.91 |
| MC10 | 99.59 | 94.26 | 20.43 | 0.75 | 99.49 | 94.96 |
| MF1 | 99.66 | 94.68 | 19.98 | 0.75 | 99.42 | 94.26 |
| MF2 | 99.67 | 94.56 | 20.41 | 0.70 | 99.51 | 94.48 |
| MF3 | 99.45 | 94.51 | 19.01 | 0.76 | 99.46 | 91.42 |
| MF4 | 99.47 | 94.52 | 19.53 | 0.77 | 99.44 | 92.06 |
| MW1 | 99.47 | 94.53 | 21.76 | 0.74 | 99.54 | 93.94 |
| MW2 | 99.52 | 94.36 | 15.70 | 0.75 | 99.31 | 86.12 |
| MW3 | 99.62 | 94.45 | 16.70 | 0.74 | 99.45 | 89.01 |
| MW4 | 99.67 | 94.65 | 20.75 | 0.70 | 99.44 | 94.83 |
| SK1 | 96.00 | 92.66 | 16.53 | 1.77 | 98.58 | 86.32 |
| SK2 | 97.90 | 92.43 | 16.71 | 1.81 | 98.56 | 86.61 |
| RD1 | 99.57 | 93.07 | 19.93 | 1.81 | 98.71 | 92.84 |
| RD2 | 99.52 | 92.88 | 17.29 | 1.80 | 98.63 | 89.32 |

**Supplementary Figure 2**


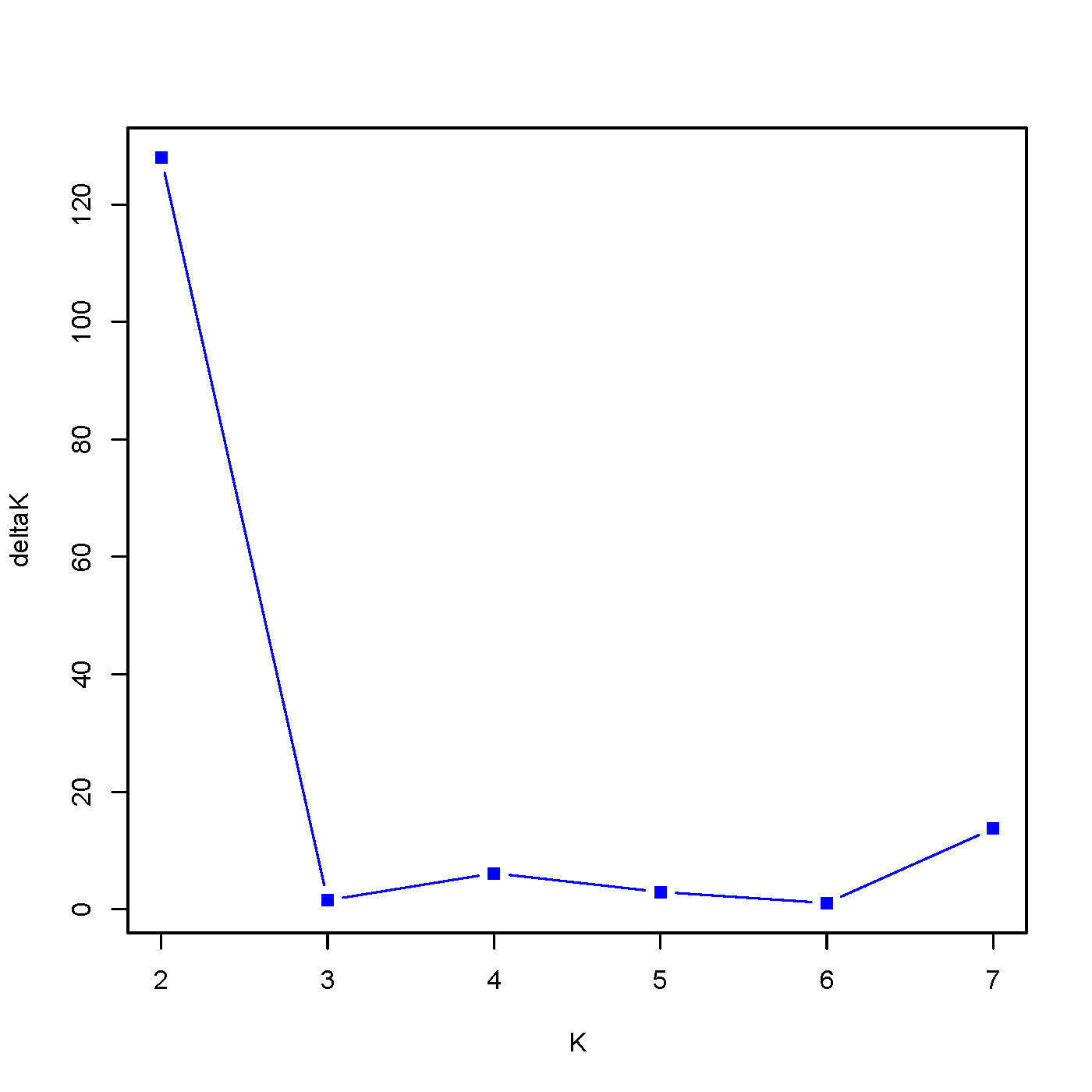

Supplement: Supplementary file 1 [file Data_Sheet_1.docx]
